# Ambient Air Pollution Increases the Risk of Cerebrovascular and Neuropsychiatric Disorders through Induction of Inflammation and Oxidative Stress

**DOI:** 10.3390/ijms21124306

**Published:** 2020-06-17

**Authors:** Omar Hahad, Jos Lelieveld, Frank Birklein, Klaus Lieb, Andreas Daiber, Thomas Münzel

**Affiliations:** 1 Center for Cardiology–Cardiology I, University Medical Center of the Johannes Gutenberg-University Mainz, 55131 Mainz, Germany; omar.hahad@unimedizin-mainz.de; 2 German Center for Cardiovascular Research (DZHK), Partner Site Rhine-Main, 55131 Mainz, Germany; 3 Atmospheric Chemistry Department, Max Planck Institute for Chemistry, 55128 Mainz, Germany; jos.lelieveld@mpic.de; 4 Climate and Atmosphere Research Center, The Cyprus Institute, Nicosia 1645, Cyprus; 5 Department of Neurology, University Medical Center of the Johannes Gutenberg-University Mainz, 55131 Mainz, Germany; frank.birklein@unimedizin-mainz.de; 6 Department of Psychiatry and Psychotherapy, University Medical Center of the Johannes Gutenberg-University Mainz, 55131 Mainz, Germany; klaus.lieb@unimedizin-mainz.de; 7 Leibniz Institute for Resilience Research, 55122 Mainz, Germany

**Keywords:** air pollution, particulate matter, cerebrovascular disorders, neurological disorders, mental disorders, stroke, dementia, oxidative stress, inflammation

## Abstract

Exposure to ambient air pollution is a well-established determinant of health and disease. The Lancet Commission on pollution and health concludes that air pollution is the leading environmental cause of global disease and premature death. Indeed, there is a growing body of evidence that links air pollution not only to adverse cardiorespiratory effects but also to increased risk of cerebrovascular and neuropsychiatric disorders. Despite being a relatively new area of investigation, overall, there is mounting recent evidence showing that exposure to multiple air pollutants, in particular to fine particles, may affect the central nervous system (CNS) and brain health, thereby contributing to increased risk of stroke, dementia, Parkinson’s disease, cognitive dysfunction, neurodevelopmental disorders, depression and other related conditions. The underlying molecular mechanisms of susceptibility and disease remain largely elusive. However, emerging evidence suggests inflammation and oxidative stress to be crucial factors in the pathogenesis of air pollution-induced disorders, driven by the enhanced production of proinflammatory mediators and reactive oxygen species in response to exposure to various air pollutants. From a public health perspective, mitigation measures are urgent to reduce the burden of disease and premature mortality from ambient air pollution.

## 1. Introduction

**Figure 1:**
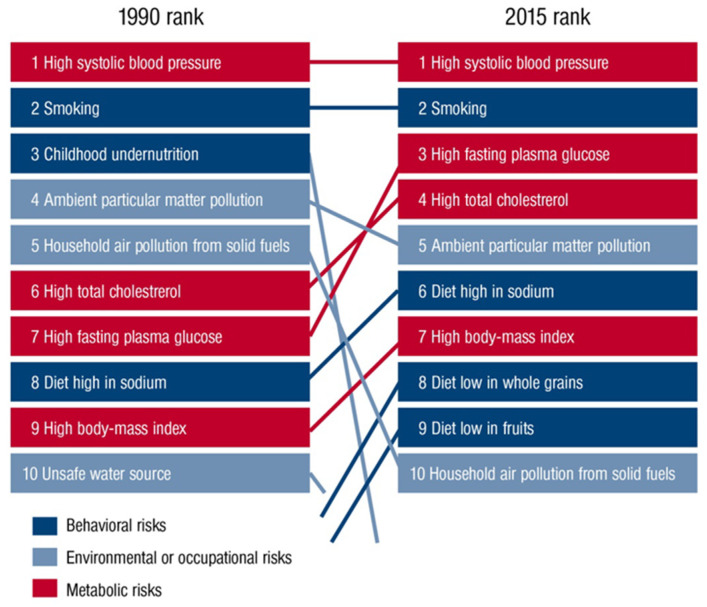
Global risk factors for global deaths in 1990 compared with 2015 outlined in the Global Burden of Disease Study (GDB). Reused from Münzel et al. [10] with permission. Copyright © 2020, Oxford University Press.

The role of environmental pollutants as an important determinant of health is being increasingly recognized. As recently outlined by the Lancet Commission on pollution and health, air pollution is the leading environmental cause of disease and premature death [1]. In this setting, diseases caused by all forms of pollution annually account for 16% of global deaths, representing 15 times more deaths than from all wars and other forms of violence as well as three times more than from AIDS, tuberculosis, and malaria combined. Likewise, the World Health Organization (WHO) concludes that 12.6 million premature deaths per year are attributable to unhealthy environments. 8.2 million of them are due to noncommunicable diseases, with cardiovascular disorders (including stroke) being here the largest contributor to the health burden, accounting for nearly 5 million of these deaths [2]. Among all environmental stressors, air pollution is the most important risk factor and ambient outdoor air pollution due to particulate matter < 2.5 µm (PM2.5) exposure ranks on the fifth position among all global health risk factors in 2015, leading to 4.2 million deaths annually (Figure 1) [3]. This is further supported by recent data from the WHO, suggesting that 9 out of 10 people worldwide breathe polluted air [4]. We recently used a novel hazard ratio function, the estimate of Global Exposure-Mortality Model (GEMM), to calculate 8.79 million global premature deaths in 2019 as well as 790,000 excess deaths per year in Europe only due to exposure to air pollution (mostly PM2.5), thereby indicating that the premature death estimates are increasing over the years [5]. However, besides being a leading cause of the global burden of noncommunicable diseases, including cardiovascular diseases, respiratory diseases, metabolic diseases, and cancer, recent studies indicated the adverse effects of air pollutants, especially of the ultrafine fraction of PM2.5, on the central nervous system (CNS) and brain health [1,6]. In this context, ultrafine particles (<0.1 µm) can translocate from the pulmonary system to the CNS by crossing the blood–brain barrier (BBB) and, ultimately, reach the brain, inducing pathophysiological alterations in the CNS due to the physical characteristics of the particle itself (relatively large reactive surface) or by toxic compounds that are bound to the particles. These mechanisms might contribute to the development of cerebrovascular and neurological disorders such as stroke, dementia, and Parkinson’s disease. Increasing evidence suggests neuroinflammation and cerebral oxidative stress to be key factors in the relationship between air pollution and cerebrovascular and neurological disorders [7], driven by the enhanced production of proinflammatory mediators and reactive oxygen species (ROS) in response to exposure to air pollutants [8,9]. Here, we provide an updated overview of the impact of air pollutants on cerebrovascular and neurological and mental disorders, along with pathophysiological insight from human and animal studies centered on inflammatory and oxidative stress pathways.

## 2. Air Pollution Mixtures and Sources

**Figure 2:**
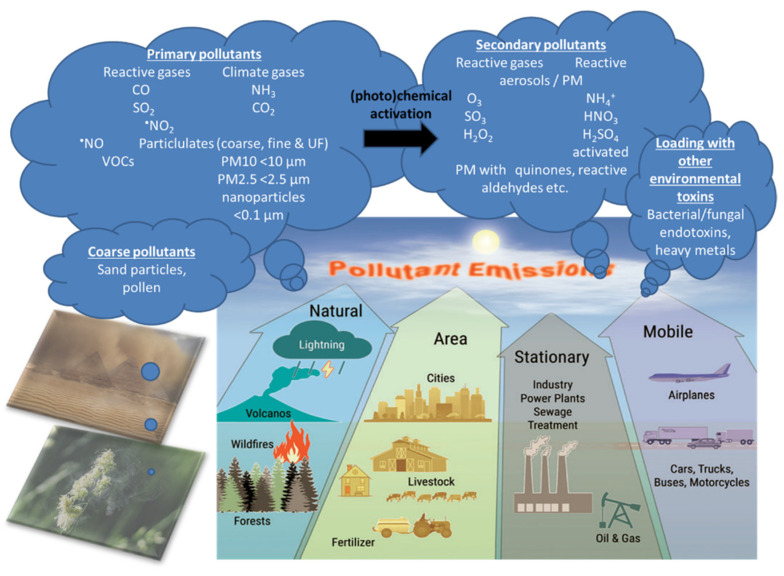
There are four main types of air pollution sources including natural, area, stationary, and mobile sources producing PM0.1, PM2.5, PM10, reactive gases including volatile organic compounds (VOCs). Primary pollutants (the indicated gases and solid particles) may undergo further toxification in the environment, e.g., by photochemical reactions by UV light producing more reactive gases or more toxic carbohydrate products on the particle surface (termed particle “aging”) [12] as well as loading of the particles with heavy/transition metals and bacterial/fungal endotoxins, leading to secondary biological toxicity [15,16,17]. The majority of coarse particles come from sediments (desert sand) and pollen from plants. Modified from Münzel et al. [18] with permission. Copyright 2020, Mary Ann Liebert, Inc., publishers. Open access source for sandstorm and plant pollen images can be found at Pixabay (https://pixabay.com/de/).

Air pollution is a heterogeneous mixture of various constituents resulting from the complex interaction of multiple emissions and chemical reactions. This mixture comprises solid particles and liquid droplets suspended in the air, i.e., PM2.5, that can include organic carbon (OC), elemental or black carbon (EC), nitrates, sulfates, and metals (e.g., iron, vanadium, nickel, copper, and manganese) as well as gases (e.g., ground level ozone (O_3_), carbon monoxide (CO), sulfur dioxide (SO_2_), oxides of nitrogen (NO_x_)) gaseous organic compounds (e.g., non-methane volatile organic compounds (VOCs), polycyclic aromatic hydrocarbons (PAHs) and polychlorinated biphenyls (PCBs)), bacterial endotoxins (mostly bound to solid particles or liquid aerosols) [11]. In the urban environment, over 90% of the pollutant mass is from gases or vapor-phase compounds, while anthropogenic combustion-derived particles are of special concern from a public health perspective due to their potential systemic toxicity owing to features such as high particle counts, high surface area to mass ratio, inflammatory and oxidative stress potential, and insoluble components, leading to facile distal airway and systemic penetration [11]. Types of atmospheric particles include coarse particles with an aerodynamic diameter between 2.5 and 10 µm (PM10), fine particles with a diameter less than 2.5 µm (PM2.5), and ultrafine particles with a diameter less than 0.1 µm (PM0.1), interconnected with the general notion that smaller particles may be more potent in inducing adverse health effects than larger particles [10]. There are four main types of sources of air pollution with fossil fuels and biomass combustion, and industry, agriculture, and wind-blown dust are also predominant sources of fine particulates in the air (Figure 2). Furthermore, it is important to note that, besides emission intensities related to e.g., the amount of fuel combusted, the number of animals in animal husbandry, industrial production levels, and distances traveled or similar activity data, air pollution is strongly influenced by climate and weather conditions [12]. For instance, factors such as wind direction and speed, atmospheric stability, and solar radiation are important determinants of the spatial (fine particles can travel more than 100 km from their place of generation) and temporal variation in air pollutants with some of the most aggressive of them being generated during hot periods with a high UV index [12]. Interestingly, air pollution and climate change influence each other via complex interactions in the atmosphere, both of which, in turn, affect public health [13]. Herein, increasing levels of pollutants such as sulfate and O_3_ can modify the energy balance of the atmosphere and earth’s surface, leading to climate change that alters the physical and chemical state of the atmosphere [14].

## 3. Pathophysiology of Air-Pollution-Induced Disorders

Since cardiovascular risk factors and diseases are triggered to a large amount by air pollution and impact a high proportion of global deaths, e.g., by inducing noncommunicable diseases, great efforts were made to explore, understand, and prevent the adverse cardiovascular effects of sustained exposure to air pollutants. On the basis of the Global Exposure-Mortality Model (GEMM), we have even shown that air pollution is a larger contributor to global mortality (8.79 million excess deaths) than one of the most important health risk factors, namely tobacco smoking (7.2 million excess deaths attributed to tobacco smoking as estimated by the WHO [19]), with a population average loss of life expectancy of 2.9 vs. 2.2 years for air pollution vs. tobacco smoking [20]. We recently reviewed the effects of gaseous and solid constituents of air pollution with a particular focus on the effect of fine particles on vascular endothelial function and clinical cardiovascular outcomes, indicating that vascular inflammation and oxidative stress are common denominators of the cardiovascular effects of air pollution [10]. Vascular endothelial dysfunction is regarded as an early subclinical key event in the development of dysregulated blood pressure and manifestation of atherosclerotic cardiovascular disease, which is not only due to classical risk factors (smoking, high cholesterol, diabetes mellitus, and hypertension) but also appears to be a consequence of environmental hazards such as air pollution [18,21]. Increasing evidence from human and animal studies suggests that exposure to ambient air pollutants leads to a pathological state of the vascular endothelium that is characterized by an imbalance between the formation and degradation of nitric oxide (^•^NO) [10]. Since the half-life and biological activity of ^•^NO as a free radical is strongly related to the existence of ROS such as the superoxide ion, reduction and decreased activity of ^•^NO as well as the direct physical damage to endothelial cells due to redox imbalance impairs several crucial functions of an intact endothelium to maintain its vasodilatory, antithrombotic, anti-inflammatory, and antioxidant effects. Thus, the persistent physiological detriments from the long-term exposure to air pollution can lead to atherosclerotic plaque formation and, over time, subsequently to various cerebro/cardiovascular disease phenotypes such as stroke, arterial hypertension, coronary heart disease, myocardial infarction, heart failure, and arrhythmia [22].

**Figure 3:**
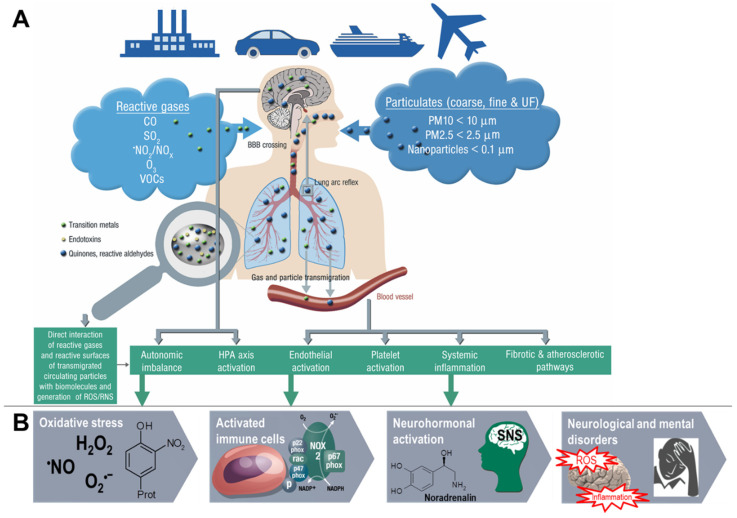
Summary of pathophysiological mechanisms by which air pollutants cause increased oxidative stress, and inflammation, thereby contributing to cerebrovascular, neurological, mental, and cardiorespiratory disorders. (**A**) Uptake and cardiorespiratory health effects triggered by air pollution constituents. (**B**) Key events that contribute to neurological and mental by air pollution constituents. Ambient PM particles are often loaded with environmental toxins stemming from particle “aging” by UV-induced photoreactions or modifications upon interaction with reactive gases in the atmosphere [12]. In addition, loading of the particles with environmental endotoxins and heavy metals enhances their direct biochemical reactivity [15,16,17]. Summarized from Münzel et al. [10] (**A**) and Daiber et al. [38] (**B**) with permission. Copyright © 2020, Oxford University Press (**A**) and © 2020 International Union of Biochemistry and Molecular Biology (**B**). SNS, sympathetic nervous system; UF, ultrafine.

Likewise, emerging evidence from human and animal studies suggests an increased risk of cerebrovascular and neuropsychiatric disorders with sustained exposure to air pollutants affecting the CNS by a variety of cellular, molecular, inflammatory, and oxidative stress pathways. However, the understanding of the underlying mechanisms remains still incomplete and complex interactions with other risk and lifestyle factors are very likely. Deeper insight into these associations is of great importance and should receive more attention, since neurological, cerebrovascular and mental disorders are among the largest causes of disability-adjusted life years and global deaths with 30% of all strokes being related to air pollution [23]. There are two possible ways by which air pollutants enter the CNS, either through direct transport of particles into the CNS or via systemic inflammation upon initial recruitment of immune cells in the lung tissue [24]. Herein, nasal inhalation and airflow constitute a direct access route in humans with the olfactory region being unique in the CNS due to its direct contact with the environmental air. Smaller particles may cross the nose-brain barrier and reach the brain via olfactory receptor neurons or the trigeminal nerve, which then can travel across the CNS and reach other brain regions. On the other hand, particles can enter the circulation via the lungs through breathing and reach the alveolar region. At this point, they can translocate to the systemic circulation through a transition process (nanoparticles probably directly, microparticles most likely via uptake by phagocytic cells and their transmigration from the lung tissue to the circulation) [25] and subsequently across the BBB to the brain parenchyma by simple diffusion or energy-dependent active transport. Once in the organism, the adverse effects of fine particulates on the brain rely mainly on three mechanisms [26]. First, they can induce the release of proinflammatory mediators leading to chronic respiratory and systemic inflammation [27], thereby affecting the BBB and ultimately triggering neural-immune interaction and resulting in increased production of ROS and chronic oxidative stress contributing to an Alzheimer phenotype in exposed children [28]. Second, the particles can damage the BBB through the direct formation of ROS and thereby alter the permeability of the barrier [29,30]. Third, there can be mechanical stimulation of specific mechano-receptors in pulmonary tissue leading to the lung arc reflex [31,32] and sympathetic activation with the release of vasoconstrictors such as catecholamines [33]. Taken together, these mechanisms are central in promoting brain inflammation, neuronal dysfunction, and neuropathology (Figure 3) (reviewed in [34,35,36,37]).

## 4. Evidence from Human and Animal Studies

Although the association between air pollution and cardiorespiratory morbidity and premature mortality is relatively well established, less is known about the effects of air pollutants on the CNS [39]. However, in recent years evidence has been accumulating from human and animal studies, suggesting a link between exposure to air pollutants, in particular fine particulates, and adverse effects on the CNS that may contribute to the development of brain diseases.

### 4.1. Human Observational/Epidemiological Studies

#### 4.1.1. Cerebrovascular Events

Among air pollution-induced disorders of the CNS, stroke is one of the most prominent disorders that have been reported to be a consequence of both short- and long-term exposure to air pollution. This relationship was first reported after the Great Smog of London, a notorious pollution episode of the 20th century, showing an increased risk of stroke deaths in response to a short-term extreme rise in air pollution [40]. However, since then, various studies emerged worldwide, pointing toward a clear association between multiple air pollutants and risk of stroke. A meta-analysis of 94 studies across 28 countries with a total of 6.2 million events found stroke hospitalization or stroke mortality to be associated with a short-term increase in levels of PM2.5 (relative risk (RR) 1.011, 95% confidence interval (CI) 1.011–1.012 per 10 μg/m^3^ increase in PM2.5), PM10 (RR 1.003, 95% CI 1.002–1.004 per 10 µg/m^3^ increase in PM10), CO (RR 1.015, 95% CI 1.004–1.026 per 1 ppm increase in CO), SO_2_ (RR 1.019, 95% CI 1.011–1.027 per 10 ppb increase in SO_2_), and NO_2_ (RR 1.014, 95% CI 1.009–1.019 per 10 ppb increase in NO_2_), indicating that daily exposure to fine particles (i.e., PM2.5) was most detrimental to stroke burden with stronger effects for ischemic than hemorrhagic stroke [41]. In good agreement, a meta-analysis of 34 studies found short-term increases in concentrations of PM2.5 (1.20%, 95% CI 0.22–2.18 per 10 μg/m^3^), PM10 (0.58%, 95% CI 0.31–0.86 per 10 μg/m^3^), CO (2.96%, 95% CI 0.70–5.27 per 1 ppm), NO_2_ (2.24%, 95% CI 1.16–3.33 per 10 ppb), O_3_ (2.45%, 95% CI 0.35–4.60 per 10 ppb), and SO_2_ (1.53%, 95% CI 0.66–2.41 per 10 ppb) to be associated with stroke admissions and stroke mortality [42]. Furthermore, a meta-analysis including 20 studies and a total of >10 million people on the association between long-term exposure to PM and stroke incidence and mortality found a hazard ratio (HR) of 1.064 (95% CI 1.021–1.109) and 1.125 (95% CI 1.007–1.256) per 5 μg/m^3^ increase in PM2.5, respectively [43]. Corresponding HRs for exposure to PM10 were 1.061 (95% CI 1.018–1.105, *p* < 0.05) for overall stroke events and 1.080 (95% CI 0.992–1.177, by trend) for stroke mortality per each 10 μg/m^3^ increase, also indicating that associations were stronger for North America and Europe than for Asia.

**Figure 4:**
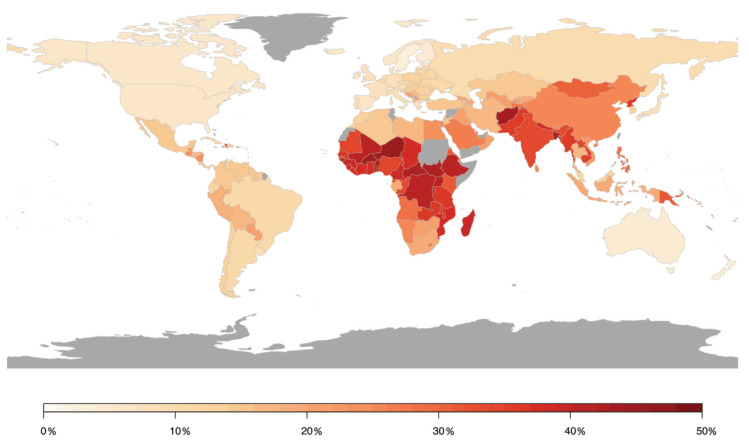
Population attributable risk of cerebrovascular disease associated with air pollution worldwide. Estimates from Institute for Health Metrics and Evaluation (IHME). Reused from Lee et al. [55] with permission according to the terms of the Creative Commons Attribution Non-Commercial License (http://creativecommons.org/licenses/by-nc/4.0/).

In contrast, a meta-analysis including 45 studies on the impact of short-term changes in levels of PM revealed inconclusive non-significant associations for hospital admissions for total cerebrovascular disease or ischemic or hemorrhagic stroke. PM2.5 and PM10 were associated with only a 1.4% (95% CI 0.9–1.9) and 0.5% (95% CI 0.3–0.7) higher total cerebrovascular disease mortality, respectively [44]. HRs of 1.11 (95% CI 1.05–1.17) and 1.11 (95% CI 1.05–1.17) for incidence of stroke and for stroke mortality were found in response to PM2.5 exposure per 5 μg/m^3^ increment, respectively, in a meta-analysis including 16 studies with more than 2.2 million people [45]. Stronger effects were found in North America and Europe compared to Asia. These results were confirmed in recent studies from Asia and Europe. A meta-analysis analyzing 8,359,162 hospital admissions due to stroke events in 248 Chinese cities has shown that a 10 μg/m^3^ increase in PM2.5 concentration was associated with a 0.19% (95% CI 0.13–0.25), 0.26% (95% CI 0.17–0.35), and 0.26% (95% CI 0.13–0.38) increase in same-day hospital admissions for total cerebrovascular disease, ischemic stroke, and transient ischemic attack, respectively, whereas no substantial association was observed for hemorrhagic stroke [46]. Additional adjustment for SO_2_, NO_2_, CO, and O_3_ did not alter these associations. Likewise, a prospective cohort study from China (*n* = 117,575) revealed that long-term residential exposure to PM2.5 increased the risk of incident stroke, ischemic stroke, and hemorrhagic stroke by 13% (HR 1.13, 95% CI 1.09–1.17), 20% (HR 1.20, 95% CI 1.15–1.25), and 12% (HR 1.12, 95% CI 1.05–1.20), respectively, for each increase of 10 μg/m^3^ [47]. In a Vietnamese study adjusting for meteorological factors, indicators of holidays and influenza epidemics, multiple air pollutants were found to increase daily hospital admissions due to a variety of cardiovascular conditions, including hospitalizations for stroke in response to elevated levels of SO_2_ [48]. Recent data from India based on the analyses of 29 Indian cities (with at least 1 Mio. inhabitants), one of the most polluted countries in the world, being the fifth-highest-ranking country for PM2.5 pollution in 2019, indicated that stroke was the second leading cause of premature deaths attributable to PM2.5 exposure, accounting for 22%. Ischemic heart disease is the first leading cause of premature deaths attributable to PM2.5 exposure accounting for 58% [49]. In two prospective studies from Sweden and Spain, no association was found between long-term exposure to PM2.5 and/or PM10 and incident stroke events, which may have been influenced by the comparatively low exposure levels [50,51]. A large prospective study from Canada (*n* = 5,071,956) found increased HRs of incident stroke for long-term exposure to PM2.5, NO_2_, O_3_, and O_x_, with risk increases of 4–5% after adjustment for individual- and neighborhood-level variables [52]. Conversely, results from the Women’s Health Initiative, a large prospective study from the US, displayed that short-term exposure to PM2.5, PM10, NO_2_, NO_x_, SO_2_, and O_3_ was not associated with risk of total stroke, ischemic stroke, or specific etiologies of ischemic stroke, whereas NO_2_ (odds ratio (OR) 1.24, 95% CI 1.01–1.52 per interquartile range (IQR) increase) and NO_x_ (OR 1.18, 95% CI 1.03–1.34) were associated with increased risk of hemorrhagic stroke in post-menopausal women [53]. The role of short-term PM2.5 concentrations for fatal hemorrhagic stroke was examined in a Chinese study showing that risk was particularly pronounced in subjects with diabetes mellitus (OR 1.26, 95% CI 1.09–1.46), emphasizing that subjects with preexisting disease conditions may be more susceptible to the adverse effects of air pollution [54]. A summary of the association between air pollution and cerebrovascular disease (mostly stroke) is provided in Figure 4, presenting the relative risk for the development of cerebrovascular disease in association with air pollution by country [55].

#### 4.1.2. Dementia

The role of fine particulate matter as a potential determinant of dementia was analyzed in two recent meta-analyses. The meta-analysis of four cohort studies from Canada, Taiwan, the UK, and the US including > 12 million elderly subjects aged ≥ 50 years reported more than a 3-fold increase in dementia risk (HR 3.26, 95% CI 1.20–5.31 per 10 μg/m^3^) with long-term exposure to PM2.5 [56]. Moreover, subgroup analyses revealed an almost 5-fold increased risk of Alzheimer’s disease (HR 4.82, 95% CI 2.28–7.36). A comprehensive meta-analysis with inclusion of 80 studies covering 26 countries aimed to analyze the influence of PM2.5 on a variety of cerebrovascular and neurological disorders [57]. The authors found long-term PM2.5 exposure to be associated with increased overall risk of dementia (OR 1.16 95% CI 1.07–1.26) and Alzheimer’s disease in particular (OR 3.26, 95% 0.84–12.74) along with increased risk of autism spectrum disorder (OR 1.68, 95% CI 1.20–2.34), Parkinson’s disease (OR 1.34, 95% CI 1.04–1.73), and stroke. Interestingly, two recent studies identified cardiovascular diseases as playing a crucial role in modifying and mediating the association between air pollutants and dementia risk [58,59]. In the more recent one, a prospective study from Sweden, the presence or development of heart failure, ischemic heart disease, and stroke (as the most intermediate condition explaining 49.4% of air pollution–related dementia cases) seemed to enhance the association between long-term exposure to PM2.5 and NO_x_ and risk of dementia, most likely due to shared pathophysiological pathways by which air pollutants exert adverse cardiovascular and neurological effects [58]. The authors concluded that since cardiovascular diseases accelerate cognitive decline and anticipates the onset of dementia, exposure to air pollution may negatively affect cognition by detrimental effects through cardiovascular disease, even without directly reaching the brain. In support of this, the results of a prospective study from Italy demonstrated that brain effects of air pollution are clearly linked with vascular damage, as shown by the positive long-term associations of NO_x_, NO_2_, PM2.5, and PM10 and vascular dementia, whereas relationships with Alzheimer’s disease and senile dementia were less clear [60]. In line with this, a Taiwanese study found, after adjustment for potential confounders and other air pollutants, that PM10, CO, and NO_2_ are associated with OR of vascular dementia [61]. These relationships were confirmed in further studies from Sweden and Canada showing that exposure to multiple air pollutants may increase the risk of vascular dementia and Alzheimer’s disease [62,63,64,65]. Importantly, since traffic is not only a main source of air pollution in urbanized areas but also associated with increased noise pollution, studies have investigated the effects of air and noise pollution and risk of dementia. However, no evidence was found for an association between road traffic noise exposure and dementia risk as well as no interaction between noise and air pollutants to modulate the risk of dementia [66,67].

#### 4.1.3. Parkinson’s Disease

Evidence regarding the link between air pollution and Parkinson’s disease is limited and results appear to be generally inconclusive (e.g., as smokers with high PM exposures have lower risk for development of Parkinson’s Disease). The most recent meta-analysis on the association between long-term exposure to air pollution, second-hand smoke, and onset of Parkinson’s disease including a total of 21 studies revealed marginally (mostly not significant) increased risks in response to increased concentrations per 10 μg/m^3^ in PM2.5 (RR 1.08, 95% CI 0.98–1.19), NO_2_ (RR 1.03, 95% CI 0.99–1.07), O_3_ (RR 1.01, 95% CI 1.00–1.02), and CO (RR 1.32, 95% CI 0.82–2.11) [68]. Perhaps counterintuitively, exposure to second-hand smoke was associated with substantially decreased risk of Parkinson’s disease. Accordingly, a further meta-analysis found a slightly higher risk for the incidence of Parkinson’s disease on the basis of 15 studies, resulting in an RRs of 1.06 (95% CI 0.99–1.14) for PM2.5, 1.01 (95% CI 0.98–1.03) for NO_2_, 1.01 (95% CI 1.00–1.02) for O_3_, and 1.34 (95% CI 0.85–2.10) for CO following long-term exposure, while RR for hospital admission due to Parkinson’s disease was 1.03 (95% CI 1.01–1.05) in response to an increase in PM2.5 short-term exposure, with overall high heterogeneity between studies [69]. Stronger effect estimates for Parkinson’s Disease risk were found in a meta-analysis including 10 studies with RRs of 1.06 (95% CI 1.04–1.09) for NO_x_, 1.65 (95% CI 1.10–2.48) for CO, 1.01 (95% CI 1.00–1.03) for NO_2_, and 1.01 (95% CI 1.00–1.02) for O_3_, however, there was a high risk of bias [70].

#### 4.1.4. Cognitive Decline

The results from a number of studies suggest that chronic air pollution exposure has neurotoxic effects that culminate over time to neuronal damage and loss, leading to cognitive dysfunction as an important intermediate event in the pathogenesis of dementia, predicted to be the result of cumulative exposure across a lifetime [24]. A recent study from the US has shown that long-term exposure to higher levels of air pollutants, i.e., PM2.5, PM10, and NO_2_, were cross-sectionally and longitudinally related to pronounced cognitive decline among older adults [71]. Among elderly Taiwanese, long-term exposure to PM10 (OR 1.094, 95% CI 1.020–1.174) and O_3_ (OR 1.878, 95% CI 1.363–2.560) was related to higher odds of cognitive impairment with evidence of a joint effect of both exposures [72]. A Chinese study used the air pollution index (calculated based on daily readings of SO_2_, NO_2_, and PM10) to demonstrate that sustained lower air quality impedes cognitive performance in verbal and math tests after adjustment for a range of individual variables that was pronounced among older people, in particular for men and the less educated [73]. Conversely, a South Korean study of elderly people revealed that long-term exposure to PM10 (OR 1.01, 95% CI 1.00–1.03) and PM2.5_–10_ (OR 1.03, 95% CI 1.01–1.07) resulted in higher odds of decreased cognitive function in women compared to men [74]. Interestingly, in older US citizens it was demonstrated that long-term effects of PM2.5 on cognitive decline was especially pronounced among subjects living in high-stress neighborhoods, which implies that people living in disadvantaged neighborhoods, where social stressors and environmental hazards are more common, may be particularly susceptible to the adverse health effects of air pollution [75]. Likewise, a study of middle-aged and older US adults assessing the association of multiple air pollutants and domain-specific cognitive function using a neuropsychological battery displayed that increased exposure to PM2.5, NO_2_, and O_3_ was related to lower verbal learning, lower logical memory, and lower executive function, respectively [76]. Long-term exposure to higher concentrations of PM2.5 was shown to increase memory decline after a 5-year follow-up in older UK adults [77]. Further studies from the US have confirmed the link between higher exposure to air pollutants, i.e., PM2.5 or black carbon, with pronounced cognitive decline in older adults after adjustment for individual confounders [78,79,80].

#### 4.1.5. Headache and Migraine

Changing weather conditions are regarded as one of many triggers of headache and migraine. The direct association of headache and migraine with air pollution is less clear and only a limited number of studies to date have investigated a potential link, demonstrating mixed results. Recently, a study analyzed the relationship between weather, air pollution, and risk of migraine headache onset [81]. Higher odds of migraine headache were associated with relative humidity in the warm season (April–September) and with higher levels of daily maximum 8-h O_3_ and CO in the cold season (October–March). However, these associations were influenced by further adjustment to confounders with seasonality remaining the predominant factor for migraine headache onset. More consistent associations were found in a study from Chile, indicating that daily numbers of hospitalizations for headache were increased in response to increased concentrations of CO (RR 1.11, 95% CI 1.06–1.17), NO_2_ (RR 1.11, 95% CI 1.06–1.17), SO_2_ (RR 1.10, 1.04–1.17), O_3_ (RR 1.17, 95% CI 1.08–1.26), PM2.5 (RR 1.11, 95% CI 1.00–1.19), and PM10 (RR 1.10, 95% CI 1.04–1.15) [82]. A total of four studies from Canada have examined the relationship between multiple air pollutants and emergency department visits for migraine and headache, all of which demonstrated consistent positive associations [83,84,85,86]. This was also the case for two studies from Taiwan that could demonstrate increased risk of outpatient department visit for headache with exposure to multiple air pollutants after controlling for weather variables, day of the week, seasonality, and long-term time trends [87,88]. Based on the analysis of 22,021 emergency department visits for headache, an Israeli study showed short-term increases in temperature as well as in NO_2_ concentrations to result in increased RRs of 1.042 (95% CI 1.009–1.076) and 1.110 (95% CI 1.057–1.167), respectively [89].

#### 4.1.6. Epilepsy

In a total of three studies, a potential link between air pollution and epilepsy was investigated. Two studies from China examined the short-term effects of multiple air pollutants on hospitalization for epilepsy. In the more recent one, an IQR increase in concentrations of NO_2_ and CO on the concurrent day was associated with increased admission rate of 2.0% (95% CI 0.5–3.6) and 1.1% (95% CI 0.1–2.1), respectively [90]. This was also the case for PM2.5 increases on the previous day (1.32%, 95% CI 0.16–2.48), whereas the average concentration of seven days was associated with decreased admission rate. Consistent with this, a 10 μg/m^3^ increase of NO_2_ and SO_2_ concentrations resulted in an increase of 3.17% (95% CI 1.41–4.93) and 3.55% (95% CI 1.93–5.18) for outpatient-visits for epilepsy on the concurrent days, respectively, whereas a decrease of −0.84% (95% CI −1.58–0.09) was found in response to O_3_ [91]. Positive associations were also found in a study from Chile, with corresponding RRs per IQR concentration increases of 1.098 (95% CI 1.045–1.155) for CO, 1.100 (95% CI 1.025–1.181) for O_3_, 1.085 (95% CI 1.03–1.144) for SO_2_, 1.108 (95% CI 1.021–1.204) for NO_2_, 1.083 (95% CI 1.038–1.13) for PM10, and 1.065 (95% CI 1.002–1.132) for PM2.5, without being influenced by sex, age, and season [92].

#### 4.1.7. Neurodevelopmental Disorders

Several studies suggest that air pollution-induced neurotoxicity may disproportionally affect young individuals and the developing brain, with prenatal and perinatal exposure contributing to developmental disabilities and behavioral abnormalities [93]. In particular, various recent studies have found associations between air pollution and autism spectrum disorders, which are generally characterized by impairment in socialization and communication as well as by the presence of repetitive and unusual behaviors. Indeed, evidence emerged from three meta-analyses suggesting that air pollution exposure may contribute to the increased risk of autism spectrum disorders [94,95,96]. In the most recent one, a total of 25 studies were investigated to analyze the role of maternal exposure to air pollution and risk of autism spectrum disorders in children, resulting in ORs of 1.06 (95% CI 1.01–1.11) for PM2.5 and 1.02 (95% CI 1.01–1.04) for NO_2_ [96]. In a large Canadian cohort of 132,256 births, maternal exposure to NO was associated with increased risk of development of autism spectrum disorders in children (OR 1.07, 95% CI 1.01–1.13 per IQR increase), whereas associations were less pronounced (or only showed a trend) for PM2.5 (OR 1.04, 95% CI 0.98–1.10) and NO_2_ (OR 1.06, 95% CI 0.99–1.12) [97]. Interestingly, a recent study in the US has shown that gestational diabetes mellitus and maternal exposure to O_3_ were associated with additive effects on autism risk, likely due to shared pathways that include inflammation and oxidative stress [98]. Increased risk of autism spectrum disorders in response to maternal exposure to air pollution was also found in a recent study in Sweden indicating an OR of 1.40 (95% CI 1.02–1.93) with increased NO_x_ exposure (top quartile), whereas no substantial associations were found for the risk of developing attention deficit hyperactivity disorder [99]. Moreover, there are data from Denmark showing that air pollution exposure in early infancy but not during pregnancy increases the risk of being diagnosed with autism and spectrum disorder [100]. This was confirmed by an Israeli study resulting in higher odds for postnatal exposure to NO_2_ and autism risk than for prenatal exposure [101]. With regard to this aspect, mixed results were achieved in other studies [102,103,104,105].

#### 4.1.8. Mental Disorders

Since inflammation and oxidative stress are major features of various mental disorders and given the potential of air pollution to induce such processes, investigating the association between air pollution and these outcomes is of special interest. Indeed, there is growing and substantial evidence that supports the notion that air pollution may contribute to depression, anxiety disorders, suicidal behavior, and psychoses. A recent meta-analysis including a total of 9 studies demonstrated that long-term exposure to fine particulates was associated with increased odds of depression (OR 1.102, 95% CI 1.023–1.189 per 10 μg/m^3^ increase in PM2.5), anxiety disorders (evaluated on the basis of two primary study results [106,107]), and suicide (RR 1.02, 95% CI 1.00–1.03 per 10 μg/m^3^ increase in PM10) [108]. In good agreement, a meta-analysis of 14 studies found increased odds of depression (OR 1.19, 95% CI 1.07–1.33) and suicide (OR 1.05, 95% CI 0.99–1.11] per 10 μg/m^3^ increase in PM2.5, whereas no associations were found for PM10 exposure [109]. Multiple air pollutants, including long-term exposure to PM2.5 and short-term exposure to PM10, NO_2_, SO_2_, and CO were shown to increase the risk of depression, as evaluated by a meta-analysis of 15 studies, while no evidence was found for an association between exposure to O_3_ and depression [110]. In contrast, the most recent meta-analysis including data up to 2019 from 22 studies across 10 countries, demonstrated weaker associations of air pollutants and depression including long-term exposure to PM2.5 (OR 1.12, 95% CI 0.97–1.29), PM10 (OR 1.04, 95% CI 0.88–1.25), and NO_2_ (OR 1.05, 95% CI 0.83–1.34) as well as short-term exposure to PM2.5 (OR 1.01, 95% CI 0.99–1.04), PM10 (OR 1.01, 95% CI 0.98–1.04), SO_2_ (OR 1.03, 95% CI 0.99–1.07), O_3_ (OR 1.01, 95% CI 0.99–1.03), and NO_2_ (OR 1.02, 95% CI 1.00–1.04) with difficulties to interpret the results due to high heterogeneity between studies [111]. A potential link between air pollution and psychotic experience/schizophrenia was established in two studies. A prospective study from the UK was able to demonstrate increased odds of psychotic experiences after comprehensive adjustment for confounders, which was highest among people with strongest (top quartile) long-term exposure to PM2.5 (OR 1.45, 95% CI 1.11–1.90), NO_2_ (OR 1.71, 95% CI 1.28–2.28), and NO_x_ (OR 1.72, 95% CI 1.30–2.29) [112]. The authors concluded that both biological factors such as neuroinflammation as well as psychosocial factors such as mental stress may be relevant mechanisms to explain the increased risk of psychotic experiences due to air pollution. A recent Chinese study also demonstrated an increased RR of hospitalizations due to schizophrenia (1.10, 95% CI 1.01–1.18 per IQR increase) due to increased short-term exposure to NO_2_ [113].

### 4.2. Animal Experimental Studies

As shown in Table 1, experimental data support a detrimental role of air pollution in the development of stroke with typical features that are also observed in humans (e.g., neuronal loss in the cerebral infarct volume, neuroinflammatory markers). Activation of microglia and astrocytes was also observed in air pollution-induced stroke-like conditions or aggravation of stroke models. Induction of a cerebral ischemia-like phenotype was reported for solid particles (different kinds of fine particulate matter) as well as for reactive gases. As summarized in Table 1, the impact of air pollution constituents on dementia (e.g., Alzheimer’s disease progression) is supported by a number of animal studies (reviewed in [114]). The animals showed more pronounced features or accelerated development of typical hallmarks in response to particulate matter or nitrogen dioxide exposure. The induction of a Parkinson’s disease-like phenotype by particulate matter exposure was also shown in animals, as well as the development of cognitive deficits and memory impairment in response to exposure to air pollution constituents (reviewed in [115]). In general, air pollution affects a number of vital processes in the brain such as impaired neurotransmitter signaling, higher levels of cerebral cytokines, activation of neuronal immune cells and disruption of the BBB as well as higher oxidative stress levels (indicated by oxidized low density lipoprotein) (Table 1). The above-described impairment of neuronal function and processes by air pollution is also likely to contribute to neurodevelopmental disorders (reviewed in [116,117]). The observed lateral ventricle dilation (=ventriculomegaly) in exposed animals is a typical hallmark of other poor neurodevelopmental outcomes, autism and schizophrenia.

**Figure 5:**
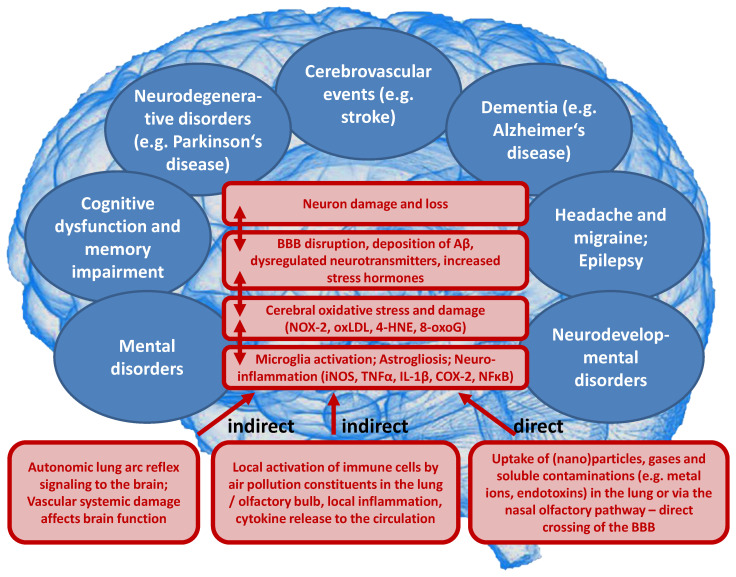
Proposed concept how air pollution constituents contribute to neurological and mental disorders. Uptake of fine particulate matter, reactive gases or secondary environmental toxins (bound to the solid or liquid aerosol particles) such as heavy metals, endotoxins or photoreaction/atmospheric chemistry products (e.g., reactive aldehydes, nitrated VOCs) by three major pathways (indirect or direct). Neuroinflammation and cerebral oxidative stress by microglia activation impairs vital pathways in the brain initiating pathophysiological processes such as amyloid deposition and neuron damage and loss. BBB, blood-brain barrier; iNOS, inducible nitric oxide synthase; TNFα, tumor necrosis factor alpha; IL-1β, interleukin 1beta; COX-2, (inducible) cyclooxygenase 2; NFκB, nuclear factor ‘kappa-light- chain-enhancer’ of activated B-cells; NOX-2, NADPH oxidase isoform 2 (phagocytic NADPH oxidase); oxLDL, oxidized low density lipoprotein; 4-HNE, 4-hydroxynonenal; 8-oxoG, 8-hydroxyguanosine; Aβ, amyloid beta peptide.

On a mechanistic level, it seems that the development of brain dysfunction in response to the exposure of animals to air pollution constituents largely depends on neuroinflammatory processes as well as cerebral oxidative stress induced by the particles or reactive gases (reviewed in [34,35,36,37]). According to the “Neuroinflammation Hypothesis”, microglia activation by direct and indirect adverse pathways induced by air pollution plays a central role [34,37]. Dysregulated microglia are central to neurotoxicity [143] by releasing neurotoxic cytokines (e.g., TNFα, IL-1β, and INF-γ) as well as different ROS (e.g., ONOO¯, O_2_^•^ˉ) [144]. Dysregulated microglia represent a hallmark of most neurological complications as well as some mental disorders [34,37], and adverse redox regulation of and by microglia plays a central role for these processes [145,146,147]. Microglia identify nanometer-sized DEP with the MAC-1 receptor to produce ROS [148] through NOX-2 activation [149]. The different pathophysiological processes that contribute to the neurological and psychiatric health outcomes, as well as the sequence of these events, are summarized in Figure 5.

## 5. Neurological Complications of Coronavirus Disease 2019 (COVID-19) and Air Pollution

An increasing body of evidence has supported CNS involvement in the pathophysiology of COVID-19. Although COVID-19 predominantly affects the respiratory and cardiovascular system, recent reports indicate its potential to cause specific and unspecific neurological symptoms/conditions such as headache, dizziness, hypogeusia, encephalopathy, encephalitis, acute cerebrovascular events, polyneuritis, impaired consciousness, and skeletal muscular injury, that can even precede the typical features like fever and cough [150]. Mechanistically, several pathways have been proposed by which the novel coronavirus (SARS-CoV-2) causes neurological complications including direct damage to neurons and specific receptors, cytokine-related injury, secondary hypoxia, and retrograde travel along nerve fibers [151]. However, the exact mechanisms of the neurological manifestation of COVID-19 remain largely elusive. In general, it has been suggested that neurological dysfunction may be the result of direct viral injury and/or systemic disease [152]. In this context, the virus may interact with brainstem pathways, resulting in indirect respiratory dysfunction, in addition to direct pulmonary injury. The coronavirus uses the angiotensin-converting enzyme 2 (ACE2) receptor to enter the cell and circulation. Since these receptors are also located in glial cells in brain and spinal neurons, it can attach, multiply, and damage neuronal tissue [150]. Some evidence indicates that the coronavirus, which resembles the ultrafine fraction of PM2.5_,_ can reach the brain through retrograde transport along the olfactory nerve and bulb. Viral binding on endothelial cells of the BBB through expression of ACE2 causes subsequent disruption of the BBB, facilitating viral entry into the CNS. Pulmonary viral invasion generates systemic inflammation (via increased levels of interleukin-6, -12, -15, and tumor necrosis factor alpha), leading to a proinflammatory state of the CNS via glial cell activation. Systemic and local lung alveolar effects act together to cause severe hypoxia, ultimately leading to cerebral vascular dysfunction [151]. Interestingly, a recent study examining the relationship between long-term exposure to NO_2_ and coronavirus fatality concluded that exposure to air pollution may be an important contributor to deaths related to COVID-19 [153,154]. Based on the analysis of death cases across 66 regions in Italy, Spain, France, and Germany, it was shown that almost 80% of all deaths were in five regions located in north Italy and central Spain, that also had the highest NO_2_ concentrations, combined with downwards airflow which interferes with dispersion of air pollution.

## 6. Conclusions

The present review summarizes recent studies on the association between ambient air pollution and cerebrovascular, neurological and mental disorders, clearly indicating that exposure to various air pollutants, especially fine particulate matter that can easily enter the organism, has the potential to contribute to stroke, dementia, Parkinson’s disease, cognitive dysfunction, neurodevelopmental disorders, and other related conditions. Inflammation and oxidative stress are regarded as central pathophysiological mechanisms by which air pollution induces brain damage. Although the adverse health effects induced by exposure to air pollution may be small or modest on an individual level, given the large proportion of the population that is exposed to air pollutants during a lifetime, the overall attributable burden may be considerably higher. However, since the research on the relationship between ambient air pollution and the CNS is relatively new, with the provided results being partly inconclusive or contradictory between and within studies, these discrepancies preclude definite conclusions. Differences in results may occur due to a variety of reasons that include discrepancies in the identification of the most relevant time of exposure for the outcome of interest (acute vs. chronic), assessment of air pollution exposure (in particular due to exposure measurement error related to spatial misalignment of monitoring data and study participants), measurement and definition of outcome variables, confounding (individual- and area-variables) and adjustment for co-exposure to air pollutants, and selection bias arising from selection into the sample, and the varying degree of other external factors contributing to the pathogenesis of the disorders (e.g., psychosocial stress in mental disorders). Thus, there is no doubt that more research is required to elucidate these relationships. Future research efforts should target more accurate exposure measurement, a variety of clinical and subclinical endpoints, the specific effects of certain toxic compounds relative to other constituents of PM2.5, including those that generate reactive oxygen species, and the combined cerebral effects of various air pollutants. A further priority is the identification of susceptible subpopulations that are at increased risk of air pollution-induced diseases, e.g., subjects with preexisting conditions or with genetic susceptibility. It is important to note that, unlike other risk factors, such as smoking, physical inactivity, excessive alcohol consumption, and unhealthy diets, air pollution can hardly be avoided or improved upon by lifestyle choices. This is the reason why improvements in environmental and air quality, and pollutant emissions in particular, cannot be controlled by patients and doctors, but rather by policy makers who regulate air pollution in order to protect the public from adverse health effects. In order to achieve this, an initial step in this direction would be to acknowledge and include environmental stressors such as air pollution as significant risk factors in official guidelines for prevention.

## Figures and Tables

**Table 1:** Air Pollution Effects in Animal Models of Neurological and Mental Disorders.

Studies and Major Outcomes	Ref.
*Stroke (cerebral ischemia)*	
Seasonal variation in air particulate matter (PM10) exposure-induced ischemia-like injuries in the rat brain was attributed to varying toxin (PAHs) loading of the particles	[118]
SO_2_ inhalation contributes to the development and progression of ischemic stroke in the rat brain by enhanced endothelin-1 activity and inflammation (iNOS, COX-2, and ICAM-1 mRNA) followed by activation of caspase-3 and higher cerebral infarct volume	[119]
Air pollutants (PM generated by different engines and aluminum sulfate aerosols) caused cortical selective neuronal loss, nuclear pyknosis, karyolysis and karyorrhexis as well as activation of microglia and astrocytes (also features of stroke and other neurological disease) as revealed by magnetic resonance imaging	[120]
Stroke damage is aggravated by nano-size particulate matter in mice, secondary to more pronounced DNA damage (8-hydroxyguanosine) and oxidative stress (gp91phox, p47phox) as well as higher number of inflammatory cells (CD68 and Ly6g positive)	[121]
Glutamatergic neurons in rodent models respond to nanoscale particulate urban air pollutants (PM_0.2_) in mice, suggesting additive effects of air pollution and ischemic stroke on cerebral damage	[122]
Astrocyte activation plays a role in fine particulate matter (PM2.5)-dependent aggravation of ischemic stroke in male rats	[123]
*Dementia (Alzheimer’s disease)*	
Diesel engine exhaust accelerates amyloid β42 plaque formation in the 5X Familial AD mouse model of Alzheimer’s disease, although no additive effects on spatial working memory deficits (assessed by Y-maze and X-maze tests) and markers of inflammation (IL-1β, RANTES and MCP-1) were observed	[124]
Central role of Toll-like receptor 4 for glial inflammatory responses (higher TLR4, MyD88, TNFα, and TNFR2 mRNA) to air pollution (PM_0.2_) in rats leading to a neuroinflammatory, accelerated cognitive aging and dementia-like phenotype	[125]
NO_2_ inhalation promotes Alzheimer’s disease-like progression via cyclooxygenase-2-derived prostaglandin E2 modulation, altered astrocyte and microglia function, all of which leading to deterioration of spatial learning and memory as well as aggravated amyloid β42 accumulation in wildtype C57BL/6J or Alzheimer’s disease-prone APP/PS1 mice	[126]
Neurotoxicity of diesel exhaust nanoparticles in the rat brain is associated with increased levels of pro-inflammatory cytokines, amyloid β42, reactive oxygen species, hydrogen peroxide, nitrogen oxide metabolites and apurinic/apyrimidinic sites (DNA damage)	[127]
Exposure of mice to particulate urban air pollution reduced the repressive epigenetic marks (H3K9me2/me3) and increased DNA damage (γ-H2AX) as well as Alzheimer’s disease hallmarks (hyperphosphorylated tau and amyloid-β plaques) in the brain	[128]
Traffic-related air pollutants (nano-sized PM) promote neuronal amyloidogenesis (amyloid-β deposition) through oxidative damage (4-HNE, 3NT) in lipid rafts of mice	[129]
*Parkinson’s disease*	
Developmental exposure to concentrated ambient ultrafine particle air pollution (similar to the paraquat and maneb model) cause a Parkinson’s disease phenotype in male mice with locomotor dysfunction and dopaminergic and glutamatergic changes	[130]
*Cognitive and memory impairment*	
Selective memory and behavioral alterations after ambient ultrafine particulate matter exposure (using the Harvard ultrafine concentrated ambient particle system) in aged 3xTgAD Alzheimer’s disease mice	[131]
Developmental exposure to low level concentrated ambient ultrafine particle air pollution and cognitive dysfunction in mice revealed by complementary learning (repeated learning), memory (novel object recognition, NOR), impulsive-like behavior (differential reinforcement of low rate (DRL), schedule of reward and delay of reward (DOR)), motor activity (locomotor behavior) and motivation (progressive ratio schedule) assessment assays	[132]
Activation of NLRP3 in microglia exacerbates diesel exhaust particles-induced impairment in learning and memory in mice	[133]
Impairment of learning and memory, induction of oxidative stress and dysregulation of monoamine neurotransmitters in the brains of mice by exposure to volatile organic compounds and carbon monoxide mixtures	[134]
PM2.5, SO_2_ and NO_2_ co-exposure impairs neurobehavior and induces mitochondrial injuries in the mouse brain	[135]
Effects of diesel engine exhaust origin secondary organic aerosols on novel object recognition ability and maternal behavior in mice	[136]
Exposure to ambient dusty particulate matter impairs spatial memory and hippocampal long-term potentiation by increasing brain inflammation and oxidative stress in rats	[30]
*Mental disorders*	
Involvement of oxidative stress and mitochondrial mechanisms in air pollution (simulated vehicle exhaust)-related neurobiological impairments in rats leading to anxiety- and depression-like behavior	[137]
Ambient PM2.5 exposure caused depressive-like responses in mice through Nrf2/NLRP3 signaling pathway and altered inflammation	[138]
Psychological impact of vehicle exhaust exposure (CO, CO_2_, NO_2_) as revealed by anxiety- and depression-like behavior as well as impaired memory in rats	[139]
Ozone exposure of rats (Flinders Sensitive Line translational model) caused neurobiological oxidative stress and a depression-like phenotype	[140]
*Other adverse effects on the brain (e.g., impaired BBB)*	
Early postnatal exposure to ultrafine particulate matter air pollution leads to dysregulated CNS neurotransmitters, cytokines and glial activation preferentially in male mice. In addition, lateral ventricle dilation (=ventriculomegaly) was observed in exposed male mice, which is associated with poor neurodevelopmental outcome, autism, and schizophrenia	[141]
Exposure to traffic-generated air pollutants mediates alterations in brain microvascular integrity (disrupted blood-brain barrier) and enhanced oxidized low density lipoprotein signaling in wildtype mice on a high-fat diet, indicating additive adverse effects of obesity and air pollution on brain function	[142]

## Abbreviations

3NT	3-nitrotyrosine
4-HNE	4-hydroxynonenal
8-oxoG	8-hydroxyguanosine
Aβ	amyloid beta peptide
ACE2	angiotensin-converting enzyme 2
BBB	blood–brain barrier
CD68	cluster of differentiation 68—also macrosialin
CI	confidence interval
CNS	central nervous system
COVID-19	coronavirus disease 2019
COX-2	inducible cyclooxygenase (isoform 2)
DEP	diesel exhaust particles
EC	elemental or black carbon
γ-H2AX	H2A histone family member X
GBD	Global Burden of Disease
GEMM	Global Exposure-Mortality Model
gp91phox	catalytic subunit of the phagocytic NADPH oxidase (isoform 2)—also NOX-2
H3K9me2/me3	histone 3 lysine 9 di- and trimethylation (epigenetic marks)
HR	hazard ratio
ICAM-1	intercellular adhesion molecule 1
INF-γ	interferon gamma
IHME	Institute for Health Metrics and Evaluation
IL-1β	interleukin-1β
iNOS	inducible nitric oxide synthase
IQR	interquartile range
Ly6g	lymphocyte antigen 6 complex locus G6D
MCP-1	monocyte chemoattractant protein-1—also CCL2
MyD88	myeloid differentiation primary response 88 protein
NFκB	nuclear factor ‘kappa-light- chain-enhancer’ of activated B-cells
NOx	oxides of nitrogen
NOX-2	catalytic subunit of the phagocytic NADPH oxidase (isoform 2)—also gp91phox
OC	organic carbon
OR	odds ratio
oxLDL	oxidized low density lipoprotein
p47phox	regulatory subunit of the phagocytic NADPH oxidase (isoform 2)
PAHs	polycyclic aromatic hydrocarbons
PCBs	polychlorinated biphenyls
PM0.1	particulate matter < 0.1 µm (ultrafine particles)
PM2.5	particulate matter < 2.5 µm (fine particles)
PM10	particulate matter with a diameter between 2.5 and 10 µm (coarse particles)
RANTES	chemokine (C-C motif) ligand 5—also CCL5
ROS	reactive oxygen species
RR	relative risk
SARS-CoV-2	severe acute respiratory syndrome coronavirus 2
SNS	sympathetic nervous system
TLR4	Toll-like receptor 4
TNFα	tumor necrosis factor alpha
TNFR2	tumor necrosis factor receptor type 2
UK	United Kingdom
US	United States
VOCs	volatile organic compounds
WHO	World Health Organization
